# Application of Optimal Designs to Item Calibration

**DOI:** 10.1371/journal.pone.0106747

**Published:** 2014-09-04

**Authors:** Hung-Yi Lu

**Affiliations:** Department of Statistics and Information Science, Fu Jen Catholic University, New Taipei City, Taiwan; University of Central Greece, Greece

## Abstract

In computerized adaptive testing (CAT), examinees are presented with various sets of items chosen from a precalibrated item pool. Consequently, the attrition speed of the items is extremely fast, and replenishing the item pool is essential. Therefore, item calibration has become a crucial concern in maintaining item banks. In this study, a two-parameter logistic model is used. We applied optimal designs and adaptive sequential analysis to solve this item calibration problem. The results indicated that the proposed optimal designs are cost effective and time efficient.

## Introduction

Computerized adaptive testing (CAT) has received much attention over the past 2 decades. Recently, CAT has become increasingly critical and has been applied to numerous standardized tests, such as the Graduate Record Examinations (GRE) test, the Graduate Management Admission Test (GMAT), and the Test of English as a Foreign Language (TOEFL). In conventional paper-and-pencil testing, all examinees are presented with the same set of items. In adaptive testing, an individual set of test items, rather than a common set of test items, is given to a particular examinee. The items that constitute the individual sets are selected from an item pool according to information regarding the ability of the examinee, which is obtained during the testing process, and the test proceeds until several information criteria are satisfied. In CAT, items can be adaptively selected using the assistance of high-speed computing technology according to the optimal set of criteria for estimating the latent trait levels of the examinee. CAT can provide more efficient estimates of examinees’ latent trait levels by reducing testing time and maintaining a high level of estimate precision [1]–[4].

The item pool used in CAT is a collection of items that have been calibrated to enable the routine testing of examinees. The items chosen for an examinee in CAT are adaptively based on the responses of the examinee to previously administered items. Thus, items are selected sequentially during the course of the test. Certain item selection procedures can yield more accurate estimates and are more efficient than random selection based on testing time (test length), and numerous item selection procedures have been proposed [5]–[7]. Empirical studies have demonstrated that using item selection procedures in which Fisher information is maximized results in the overexposure of items with high discrimination and the underexposure of those with low discrimination [8], [9]. Because examinees participating in CAT are presented with various sets of items drawn from an item pool, the attrition speed of the items is extremely fast compared with that of traditional tests; therefore, replenishing the item pool is essential in CAT. To replace the previous items with new items, calibrating the item parameters of the new items is necessary. In addition to education studies, in sociology and psychology, researchers usually use questionnaires. After the aim of the study are decided, researchers need to estimate the parameters for each question, which means item calibration, and then researchers can design the questionnaires based on the aim of the study. With the different aims of studies and the changes of the society, we have to introduce new questions to meet the researching requirements; that is, calibration is a process of setting a measuring device in order to conform with a reference standard. Therefore, item calibration is an important issue in sociological and psychological researches. This causes the problem of item calibration to occur, which involves estimating item parameters based on item response models before adding the items to the item pool. This subsequently prompts the concern as to how examinees are selected based on the new items, which is typically an extremely expensive and time-consuming process [10], [11]. The problem of item calibration involves selecting examinees for new items. Online calibration is commonly used to calibrate new items. Online calibration refers to estimating the parameters of new items through active testing by presenting new items to examinees during the course of a test designed to estimate their latent trait levels. In other words, the latent trait levels used for calibrating new items are selected and estimated during an operational test.

The optimality problem involves choosing the desired values of variables for estimating the unknown parameters. Several optimal criteria, such as A-, D-, and E-optimality, have been proposed in the literature. In linear models, optimal designs are independent of the parameters of interest, but in nonlinear models, the optimal designs typically depend on the unknown parameters [12]–[15]. Sequential or multistage procedures can be used to solve the problem of unknown item parameters [16]–[19].

The most commonly applied theory in standardized testing is the item response theory (IRT). IRT is a psychometric model that describes the item characteristic curve (ICC), which is the probability of an examinee answering a particular item correctly, given a latent trait level and the parameters of the item. Several IRT models have been developed using psychological and educational measurements, such as the latent linear [20], normal ogive [21], and logistic models [22]–[26]. Among these models, logistic-type models are the most often used. IRT models are typically nonlinear, and the optimal design depends on the unknown parameters of interest. Consequently, no fixed sample size procedure is available for achieving the optimal design without acquiring further information regarding the unknown parameters. The sequential method is the most commonly used statistical method for both providing the optimal design and controlling estimation accuracy [27]. Item selection is essential in designing a test, and in this study, we reversed the perspective of item parameters and latent traits. The item calibration problem involves estimating the item parameters of given items by administering these items to the selected examinees with known latent trait levels. However, inviting additional examinees to participate in the item calibration increases the cost of calibration. In this paper, several optimal designs for item calibration are discussed, and the performance of these designs is evaluated based on estimation accuracy and efficiency regarding the number of examinees used for calibration such that the item parameter estimate can achieve the prefixed accuracy.

### Optimal Designs Used in Item Calibration

The logistic model is one of the most commonly used models for analyzing binary response data. It describes the relationship between a dichotomous response variable *Y* and a set of explanatory variables *X* according to
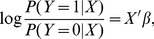
(1)which implies that



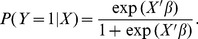
(2)Consider 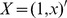 and 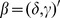; a logistic model for an explanatory variable *x* can be written as.
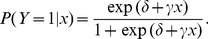
(3)


A sampling design for logistic models contains a vector of *m* design points 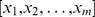 and the corresponding sample sizes 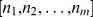. The sample size of the design is equal to 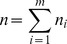 and 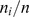 is replaced with 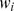 to obtain 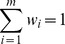. Thus, the design can be described as 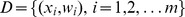. Therefore, the information matrix for the joint estimation of *δ* and *γ* is
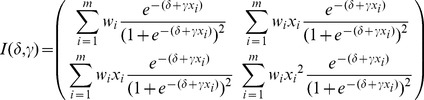
(4)


The design problem that subsequently occurs depends on the unknown parameters of interest 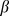. Specifically, the Fisher information matrix of 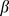 depends on both the design *X* and the unknown parameter 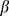.

### Item response theory models

Item response theory models describe the probability of an examinee answering a particular item correctly, given a latent trait level and the parameters of the item. Logistic models are the most frequently used models. A three-parameter logistic model (3-PL model) is formulated as
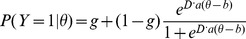
(5)where the response *Y* = 1 or 0 denotes that whether the answer is correct or incorrect, respectively. The notation *D* is a constant (for convenience, we assumed *D* = 1 in this study), and parameters *a, b,* and *g* are designated as discrimination, difficulty, and pseudo guessing parameters respectively. If *g* = 0, it is called a two-parameter logistic model (2-PL model). If all of the discrimination parameters *a* equal a fixed positive constant, or all of the items in the item bank are assumed to have the same item discrimination parameter, the logistic model becomes a Rasch model [26].

### Optimal designs for a 2-PL model

The problem of item calibration involves estimating the parameters of given items by administering these items to selected examinees with known latent trait levels. Supposing that a 2-PL model is used, to apply the results in a regular logistic regression model, several reparametrization schemes are used for convenience.

Let 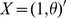 and 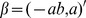; a 2-PL model can be rewritten as a regular logistic model. Thus, the item calibration process used in a 2-PL model becomes a design problem in a regular logistic model.

The optimality problem involves choosing the desired values of variables for estimating unknown parameters. Several optimal criteria, such as A-, D- and E-optimality, have been proposed in the literature [28]. Optimal design theory is widely used in educational testing, and has been developed for efficient parameter estimation [29]–[31].

#### D-optimality

Let 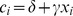 and the set 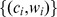 be the optimal design in this study. The criterion of the D-optimal design is to maximize the determinant of the Fisher information matrix of the parameter of interest. Mathew and Sinha showed that the symmetric design 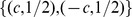 maximizing the determinant of the Fisher information matrix of 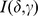, where *c* = 1.5434, is obtained by maximizing 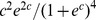 [32]. In the 2-PL model case, the design points are placed evenly on 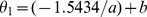 and 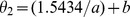, where *a* and *b* are the parameters of an item.

#### A-optimality

The A-optimal design can be obtained by minimizing the trace of the inverse of the Fisher information matrix. No explicit solution to the A-optimality problem exists under logistic models, the solution can be performed numerically [32], [33]. In the field of symmetric designs, Sitter and Wu demonstrated that the A-optimal design is obtained using 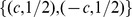 [34], where *c* minimizes
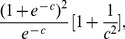
(6)where *c* can be demonstrated to be approximately 1.3 and −1.3.

#### E-optimality

The purpose of the E-optimal design is to maximize the minimum eigenvalue of the information matrix. Therefore, the problem is to identify the optimal value of *c* that is the minimization of
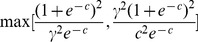
(7)


### Sequential Estimation Procedure

This section introduces the sequential optimal design procedure for item calibration. Sequential estimation has been studied by many authors [29], [35], [36]. The sequential optimal design procedure was combined with sequential estimation of parameters. The procedure is begun with an initialization phase and is complete when a stopping criterion is satisfied in the sequential estimation phase [29], [37], [38].

### Initialization phase

(1) Select an initial set of uniformly distributed design points 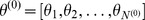 with sample size 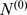, and 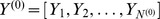 are the corresponding responses. The initial estimates a and b can then be obtained: 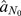 and 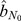. (To calibrate an item, suitable examinees must be selected to ensure that estimates of item parameters satisfied certain properties typical of a sequential design problem. Because item parameters are unknown in the initialization phase, examinees with various abilities were uniformly selected to examine and estimate item parameters. To review similar procedures, please refer to [29] and [36]).

### The *k*th iteration

(2) Compute two design points 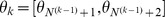 based on the previous estimates obtained from a different design scheme and their respective responses 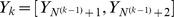. Subsequently, update the estimates of 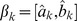 by using all of the design points 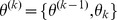 and their responses 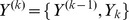.

(3) If the stopping criterion is satisfied, the procedure is stopped, and 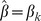 and 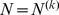. Otherwise, set 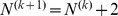, and repeat the iteration until the stopping criterion is satisfied.

#### Sequential Fixed Accuracy Estimate

In this study, we constructed a sequential confidence set for the regression parameter 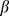 with the prescribed accuracy and precision. Chang and Martinsek considered fixed size confidence ellipsoids for parameters of a logistic regression model, and they showed that their stopping rule is asymptotically efficient when the size of the region is small [35]. Define
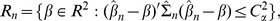
(8)where 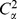 is a prefixed constant satisfying 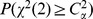, and 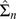 is the estimated Fisher information matrix of the true parameter 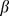. The set 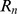 is a confidence ellipsoid of 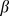 with a coverage frequency equal to 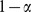, asymptotically; in other words,



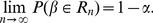
(9)If the maximum axis of 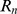 must be no greater than *2d* when *d*>0, the equivalent is obtained 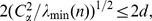 where 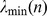 is the minimum eigenvalue of 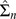. This implies that
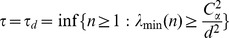
(10)for estimating 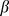. If the stopping rule 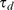 is applied, when the sampling stops, 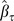 and 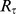 are used as the final estimate and the confidence ellipsoid of 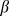, respectively. This demonstrates that 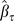 is highly consistent, and



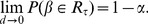
(11)Because of reparametrization, the accuracy of 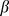 cannot be transferred to the accuracy of the item parameters of interest, *a* and *b*, directly. Therefore, because we are interested in the item parameters, rewriting the accuracy of the 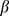 estimate based on the accuracy of the item parameters of interest is crucial. The relationship between the accuracy of 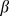 and the accuracy of the item parameters is described in the following section.

#### Accuracy of Item Parameters

Let 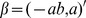, as before. As defined in Chang [39], the sequential confidence ellipsoid of 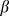 has a maximum axis no greater than 2*d* ( = *h*) and a coverage probability equal to 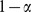, asymptotically, for a given 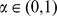 and a prescribed width *d*>0.

This implies that, at a probability equal to 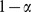,
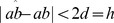
and



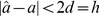
(13)Assume that *n* is sufficiently high that 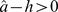, which implies that 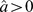. If 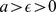 for 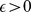, a sufficiently low *h* exists that 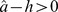 for a high *n*. This condition is mild because we assume that the discrimination parameter *a* is bounded away from 0, according to IRT.

Define 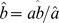. Thus, 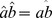. Subsequently, based on (12) and (13),
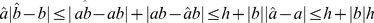



This implies that
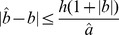
(15)


### A Simulation Study

In this study, we used a 2-PL model to describe and compare the performance of various designs. The discrimination parameter *a* ranged from 0.5 to 2.5 with an increase equal to 0.5, and the difficulty parameter *b* ranged from −3 to 3 with an increase equal to 1. Therefore, 35 combinations of item parameters were considered in the simulations.

At the initial stage, no prior information on the parameters of interest is available. Therefore, all of the possible latent trait levels should be considered. A suitable choice of design points is a set of uniformly distributed design points derived from the range of latent trait levels [−3.6, 3.6]. At the design stage, two design points are computed based on the initial estimates, and the estimates of parameters *a* and *b* are updated with the new responses. In this study, we assume that all selected latent trait levels for calibration can be specified. The sequential procedures proposed here are based on the maximum likelihood estimates. The procedure stops when the stopping criterion is satisfied. The length of the maximum axis of the confidence ellipsoid was *d* = 0.5 and the target coverage frequency was 95%. The initial sample size was 50 and each item was run 1000 times. All of the simulations were performed on an Intel personal computer, using Matlab 7.0 software.

## Results

Table 1 lists the coverage frequencies for various optimal designs. The coverage frequencies of all of the designs were over 99%, indicating that all of the cases achieved the prespecified 95% precision target.

**Table 1:** Coverage frequency of parameters.

parameter	D-optimal	A-optimal	E-optimal	Random design
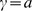	0.9990	0.9994	0.9993	0.9992
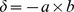	0.9925	0.9924	0.9920	0.9917

Chang and Martinsek considered fixed size confidence ellipsoids for parameters of a logistic regression model and suggested a stopping rule for constructing a confidence ellipsoid that features a “maximum axis no greater than 2*d*” and the prespecified coverage probability [35]. In other words, after stopping sampling based on this stopping rule, the errors of all parameters are smaller than 2*d*. Hence, this stopping rule is conservative and the coverage probability is typically higher than the prespecified probability. To review similar results, please refer to [36].

The original design is that of a regular logistic model with parameter 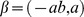, such that the estimate of 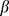 has the desired properties. However, in the 2-PL model, the item parameter is (*a*, *b*). We adopted a reparameterized form of the 2-PL model such that the design problem of the item calibration process becomes the design problem of the regular logistic model. The accuracy of the transformed item parameters *a* and *b* is obtained using (13) and (15). The results differ for various values of *a* and *b*. The simulation results are listed in Tables 2 and 3.

**Table 2:** Mean square error of 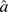.

	D-optimal	A-optimal	E-optimal	Random design
Mean square error of 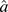 stratified by *a*				
*a* = 0.5	0.0057	0.0087	0.0113	0.0096
	(0.0012)	(0.0044)	(0.0069)	(0.0018)
*a* = 1.0	0.0091	0.0096	0.0108	0.0111
	(0.0059)	(0.0062)	(0.0082)	(0.0046)
*a* = 1.5	0.0117	0.0110	0.0106	0.0124
	(0.0093)	(0.0090)	(0.0082)	(0.0080)
*a* = 2.0	0.0139	0.0132	0.0117	0.0140
	(0.0127)	(0.0121)	(0.0103)	(0.0114)
*a* = 2.5	0.0148	0.0130	0.0135	0.0149
	(0.0135)	(0.0118)	(0.0127)	(0.0135)
Mean square error of 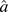 stratified by *b*				
*b* = −3	0.0041	0.0039	0.0037	0.0061
	(0.0004)	(0.0003)	(0.0003)	(0.0011)
*b* = −2	0.0068	0.0070	0.0069	0.0085
	(0.0012)	(0.0004)	(0.0008)	(0.0006)
*b* = −1	0.0142	0.0142	0.0147	0.0147
	(0.0048)	(0.0017)	(0.0013)	(0.0033)
*b* = 0	0.0278	0.0280	0.0293	0.0278
	(0.0142)	(0.0102)	(0.0068)	(0.0127)
*b* = 1	0.0139	0.0139	0.0155	0.0152
	(0.0052)	(0.0017)	(0.0016)	(0.0028)
*b* = 2	0.0067	0.0069	0.0070	0.0089
	(0.0011)	(0.0008)	(0.0006)	(0.0009)
*b* = 3	0.0040	0.0039	0.0038	0.0057
	(0.0004)	(0.0002)	(0.0003)	(0.0012)

*() standard error based on 1000 trials.

**Table 3:** Mean square error of 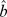.

	D-optimal	A-optimal	E-optimal	Random design
Mean square error of 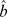 stratified by *a*				
*a* = 0.5	0.1213	0.0843	0.0498	0.1871
	(0.0291)	(0.0426)	(0.0455)	(0.0273)
*a* = 1.0	0.0179	0.0167	0.0132	0.0258
	(0.0102)	(0.0109)	(0.0106)	(0.0077)
*a* = 1.5	0.0058	0.0064	0.0078	0.0070
	(0.0046)	(0.0047)	(0.0054)	(0.0050)
*a* = 2.0	0.0020	0.0031	0.0038	0.0026
	(0.0017)	(0.0025)	(0.0030)	(0.0022)
*a* = 2.5	0.0009	0.0015	0.0019	0.0012
	(0.0008)	(0.0013)	(0.0017)	(0.0011)
Mean square error of 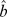 stratified by *b*				
*b* = −3	0.0184	0.0084	0.0027	0.0410
	(0.0363)	(0.0138)	(0.0022)	(0.0797)
*b* = −2	0.0300	0.0185	0.0079	0.0463
	(0.0567)	(0.0317)	(0.0087)	(0.0888)
*b* = −1	0.0375	0.0344	0.0253	0.0433
	(0.0637)	(0.0546)	(0.0365)	(0.0724)
*b* = 0	0.0389	0.0381	0.0322	0.0412
	(0.0582)	(0.0528)	(0.0374)	(0.0586)
*b* = 1	0.0375	0.0307	0.0288	0.0476
	(0.0627)	(0.0482)	(0.0441)	(0.0801)
*b* = 2	0.0266	0.0185	0.0074	0.0513
	(0.0491)	(0.0312)	(0.0077)	(0.1011)
*b* = 3	0.0182	0.0081	0.0028	0.0424
	(0.0357)	(0.0134)	(0.0024)	(0.0812)

*() standard error based on 1000 trials.

The mean square error of parameter *a* stratified according to the values of *a* and *b* is summarized in Table 2. We observed that the MSE of 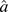 increased as *a* increased, and decreased as *|b|* increased for every design (except for the results of the E-optimal design in which the value of *a* was low). The increased *a* led to the slope of the item characteristic function to increase and the range near the true *b* to narrow; consequently, the Fisher amount of information revealed by the function decreased. Table 3 summarizes the mean square error of 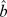. The MSE of 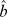 decreased as the discrimination parameter *a* increased, and decreased as *|b|* increased; thus, when discrimination parameter *a* increases, ability can be more clearly distinguished.

In summary, the parameters of the calibrated items were estimated at a prespecified precision of *d* = 0.5 and 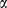 = 0.05. No significant difference occurred when estimating parameter *a* by using the various methods. In comparison with estimating parameter *b* by using these distinct methods, the precision levels for estimating parameter *b* ranked from high to low were E-optimal, A-optimal, D-optimal, and a random design when discrimination parameter *a* was low. However, when discrimination parameter a was high, the precision of estimating parameter *b* by using D-optimal and A-optimal designs was more favorable than that estimated using the E-optimal and random designs. Overall, optimal design estimations produced more precise results than random design estimations did.

**Figure 1:**
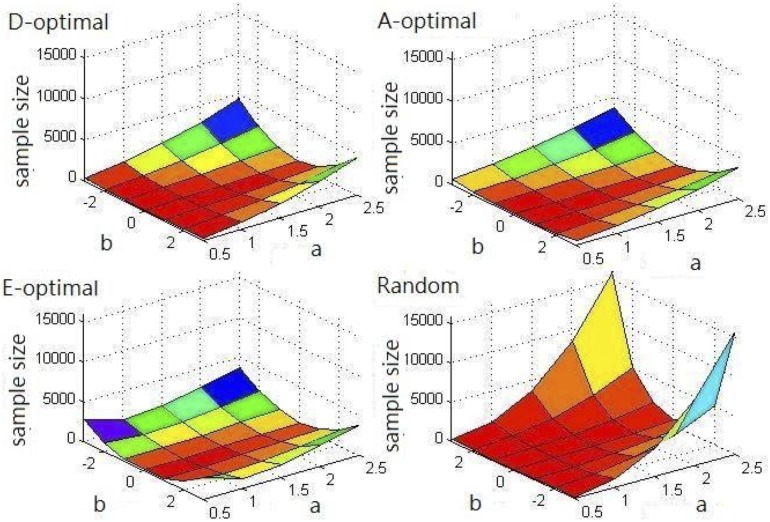
Stopping time (sample size) of items.

The estimations obtained using these four methods were not significantly different because the same stopping criterion was used. We also compared the efficiency of these four methods by determining the item calibration sample sizes. Table 4 and Figure 1 show the item calibration sample sizes of various items. When parameter *a* increased, the sample size increased. The same phenomenon occurred in |*b*|. When comparing the sample size used in the various methods, the sample size used in random design was greater than the sample size used in the other optimal designs. The reason for this is that examinees are not appropriately chosen in random designs. Therefore, less Fisher information is provided to fulfill the predefined stopping criterion. However, when parameter *a* was extremely low, the sample size used in the random designs was the smallest among the four methods because the ICC curve for random designs is flatter than the ICC curve of the other designs, and the appropriate examinee in the random designs is then chosen at a higher probability. When discrimination parameter *a* was low, the sample sizes used in the optimal designs, ranked from low to high, were D-optimal, A-optimal, and E-optimal. When discrimination parameter *a* was high, the sample sizes used in the optimal designs, ranked from low to high, were A-optimal, E-optimal, and D-optimal. Overall, the A-optimal design produced the most favorable results. The D-optimal design produced the second most favorable results, and the E-optimal design produced the least favorable results.

**Table 4:** Stopping time (sample size) of items.

	D-optimal	A-optimal	E-optimal	Random design
Stopping time stratified by *a*				
*a* = 0.5	222.7	338.5	1233.6	170.3
	(77.462)	(226.453)	(1151.069)	(45.408)
*a* = 1.0	529.3	568.1	697.0	576.1
	(339.862)	(385.647)	(495.113)	(397.618)
*a* = 1.5	941.5	885.6	891.0	1661.8
	(647.129)	(604.804)	(589.397)	(1476.011)
*a* = 2.0	1547.1	1332.0	1377.5	3631.3
	(1091.476)	(915.258)	(935.578)	(3508.672)
*a* = 2.5	2326.1	1941.2	1960.4	6601.3
	(1673.482)	(1360.741)	(1362.429)	(6508.008)
Stopping time stratified by *b*				
*b* = −3	2131.9	1939.0	2445.5	5853.2
	(1672.388)	(1226.789)	(952.1537)	(6351.605)
*b* = −2	1121.3	1015.9	1220.2	1974.5
	(861.126)	(669.150)	(510.860)	(1965.825)
*b* = −1	510.9	470.6	521.2	782.1
	(336.537)	(285.124)	(257.421)	(709.516)
*b* = 0	264.4	231.5	249.0	367.3
	(133.608)	(104.540)	(111.238)	(274.093)
*b* = 1	512.5	472.6	519.5	784.3
	(340.011)	(289.079)	(260.272)	(712.051)
*b* = 2	1121.7	1016.4	1222.5	1979.9
	(861.658)	(670.321)	(511.934)	(1966.257)
*b* = 3	2130.7	1945.6	2445.3	5956.0
	(1677.501)	(1227.314)	(955.251)	(6519.601)

*() standard error based on 1000 trials.

## Discussion and Conclusion

In CAT, the cost increases when describing a process for item calibration. Achieving correctness and efficiency in item calibration is a crucial concern. In this study, we estimated the design points for various optimal designs to discuss the accuracy and efficiency of item calibration in fully sequential analysis. Because the same stopping criterion was used for these four methods, we determined that no significant difference in the estimating parameters existed. However, the sample size used in the optimal designs was smaller than that used in random design. Furthermore, the A-optimal design produced the most favorable results compared with those of the other optimal designs.

Based on these results, we offer the following suggestions:

1. This study employed symmetric design to limit A-optimal and E-optimal, so the findings are restricted. We thus call for more future research to investigate optimal design without the assumption of symmetric design to bring more insights.
2. In this study, we assume that all selected latent trait levels for calibration can be specified. In online calibration, the latent trait levels used for calibrating new items are selected and estimated during an operational test. Thus, the selected latent trait levels for calibration are typically subject to measurement errors. For further details regarding measurement error problems in online calibration, please refer to [36].
3. In this study, we used a sequential estimation procedure. In this procedure, only two new design points are included in each iteration. This is fully sequential sampling, and the number of iterations and time required for item calibration increase. In practice, multistage sequential sampling, in which samples are selected only at several stages and the time for item calibration decreases, can be considered [40], [41].
