# Correction: Application of Optimal Designs to Item Calibration

**DOI:** 10.1371/journal.pone.0114656

**Published:** 2014-11-24

**Authors:** The PLOS ONE Staff

There is an error in the funding statement. Please refer to the correct funding statement below.

This research was supported by the National Science Council (NSC 101-2410-H-030-038-) and Fu Jen Catholic University (FJU 410031074093). The funders had no role in study design, data collection and analysis, decision to publish, or preparation of the manuscript.
